# Safety and Diagnostic Yield of Endoscopic Ultrasound-Guided Fine-Needle Biopsy for Hypervascular Pancreatic Lesions

**DOI:** 10.3390/jcm12206663

**Published:** 2023-10-21

**Authors:** Wataru Hamamoto, Takumi Onoyama, Shiho Kawahara, Yuri Sakamoto, Hiroki Koda, Taro Yamashita, Yohei Takeda, Kazuya Matsumoto, Kenichi Harada, Naoyuki Yamaguchi, Hajime Isomoto

**Affiliations:** 1 Department of Gastroenterology and Nephrology, Faculty of Medicine, Tottori University, Nishi-cho 36-1, Yonago 683-8504, Japan; hamamoto_trr@yahoo.co.jp (W.H.);; 2 Department of Gastroenterology and Hepatology, Nagasaki University Graduate School of Biological Sciences, 1-7-1 Sakamoto, Nagasaki 852-8501, Japan

**Keywords:** endoscopic ultra-sound fine-needle biopsy (EUS-FNB), pancreatic solid lesion, adverse event, diagnostic yield, hypervascular lesion

## Abstract

Endoscopic ultrasound-guided fine-needle biopsy (EUS-FNB) is a common technique for diagnosing pancreatic lesions with high accuracy and a low incidence of procedural adverse events. However, occasional adverse events, particularly bleeding, may occur. Procedures for hypervascular lesions are considered important, but their risks are unknown. We aimed to evaluate the safety and diagnostic yield of EUS-FNB for hypervascular pancreatic solid lesions. This study included 301 patients with 308 solid pancreatic lesions who underwent EUS-FNB between May 2011 and December 2018. We performed propensity-score matching to balance clinical differences between hypervascular and hypovascular lesions and analyzed 52 lesions. We compared the safety and diagnostic performance of propensity score-matched cohorts. The sensitivity, specificity, and accuracy rates of EUS-FNB for hypervascular lesions were 94.7%, 100%, and 96.2%, and those for hypovascular lesions were 80.0%, 100%, and 84.6%, respectively. There was no difference in diagnostic performance between hypervascular and hypovascular lesions. Furthermore, adverse events occurred in only one patient (pancreatitis) in the hypovascular group. There were no significant differences in the occurrence of adverse events between hypervascular and hypovascular lesions (0% vs. 3.8%, *p* = 1.000). Therefore, EUS-FNB may be safe with a high diagnostic yield, even for hypervascular solid pancreatic lesions.

## 1. Introduction

Solid pancreatic lesions include various neoplastic and non-neoplastic diseases, such as pancreatic ductal adenocarcinoma, acinar cell carcinoma, pancreatic neuroendocrine neoplasm (PNEN), solid pseudopapillary neoplasm (SPN), malignant lymphoma, metastatic pancreatic tumors, mass-forming pancreatitis, and autoimmune pancreatitis. It is essential to diagnose pancreatic lesions correctly because treatment strategies and prognoses differ.

Since its development in 1992, endoscopic ultrasound-guided fine-needle biopsy (EUS-FNB) has been commonly used for pancreatic diseases [1]. The diagnostic utility of EUS-FNB for solid pancreatic lesions has been reported previously. In a previous meta-analysis of EUS-FNB for solid pancreatic lesions, the sensitivity, specificity, and accuracy were 85–90.8%, 96.5–98%, and 91.0%, respectively [2,3]. The safety of EUS-FNB has also been reported, and the adverse event rate of EUS-FNB for solid pancreatic lesions is approximately 2.0%. Although the diagnostic yield of EUS-FNB for solid pancreatic lesions is clear, it has mainly been evaluated for pancreatic ductal adenocarcinomas. However, there are few reports on the diagnostic performance of rare pancreatic lesions, such as PNEN, SPN, metastatic pancreatic tumors, and autoimmune pancreatitis [4,5,6,7]. Moreover, the diagnostic utility and safety of EUS-FNB for hypervascular pancreatic lesions remain unclear. In terms of safety, the adverse event rates of EUS-FNB for pancreatic hypervascular lesions, especially bleeding, may be higher than those for pancreatic hypovascular lesions. A recent retrospective study reported that PNENs, which are generally hypervascular lesions, are an important factor associated with adverse events after EUS-FNB [8]. However, neither prospective nor randomized controlled studies on EUS-FNB have focused on differences in the vascularity of pancreatic lesions. In this study, we examined the diagnostic utility of EUS-FNB for solid pancreatic hypervascular lesions and adverse events, resulting from its use in comparison with solid pancreatic hypovascular lesions using propensity-score matching analysis.

## 2. Materials and Methods

### 2.1. Study Population

**Figure 1:**
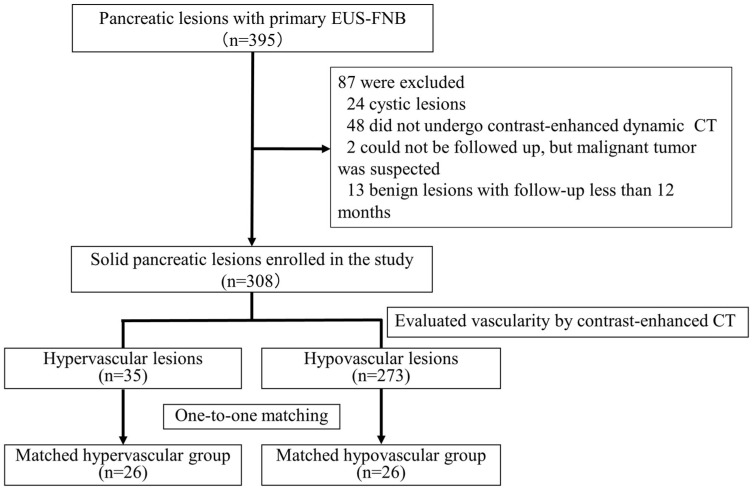
Flowchart of patients divided into matched hypervascular and hypovascular groups in the study. Abbreviations: EUS-FNB, endoscopic ultrasonography-guided fine-needle biopsy; CT, computed tomography.

We conducted a retrospective study involving patients who underwent EUS-FNB for solid pancreatic lesions at our institution between May 2011 and December 2018. The study’s inclusion criteria were as follows: (1) patients who underwent EUS-FNB for solid pancreatic lesions and (2) patients ≥20 years old who underwent endoscopic procedures. Exclusion criteria were as follows: (1) patients who underwent EUS-FNB for pancreatic cystic lesions; (2) patients who did not undergo contrast-enhanced dynamic computed tomography (CT) for pancreatic lesions; (3) patients who did not provide consent; (4) patients who received chemotherapy for malignant tumors within one month of obtaining pathological specimens; and (5) patients with benign lesions who had been followed-up for <12 months. Pancreatic lesions with a contrast enhancement greater than that of the pancreatic parenchyma in the arterial or portal phase of dynamic CT were defined as hypervascular lesions. The other lesions were considered hypovascular (Figure 1).

This study was conducted in accordance with the World Medical Association’s Declaration of Helsinki statement on the Ethical Principles for Medical Research Involving Human Subjects. The study protocol was approved by the institutional review board of Tottori University (Approval No. 18A233). Informed consent was obtained from all the participants using an opt-out approach.

### 2.2. Endoscopic Procedure

A convex-array echoendoscope, GF-UCT260 (Olympus Medical System Co., Tokyo, Japan) or EG-580UT (Fujifilm Medical Co., Tokyo, Japan) was used for the EUS-FNB. The following needles were used for the EUS-FNB: EZ shot 2/EZ shot 3 Plus (Olympus Medical System Co.), Expect/Acquire (Boston Scientific Co., Boston, MA, USA), SonoTip Pro Control (Medico’s Hirata Co., Osaka, Japan), or Echo Tip Pro Core (Cook Medical Co., Salem, NC, USA). Needles with holes were not used. The type and size of needles were selected at the discretion of each endoscopist. The proficiency of the endoscopists exhibited variability; however, when trainees conducted the procedure, they were under the supervision of a trainer. The procedures were carried out with conscious sedation. Following the delineation of the lesion and confirmation that there was no visible inflow of pancreatic duct or Doppler signal into the puncture route, punctures were executed through the gastric or duodenal wall. Contrast-enhanced EUS was not conducted before the puncture. A stylet was inserted, and the puncture was performed with the stylet in place. Subsequently, the stylet was removed from the needle, and negative suction was applied using a 20 mL syringe. A total of twenty strokes per EUS-FNB puncture were administered using the door-knocking technique [9]. To prevent tissue desiccation, specimens were promptly immersed in formalin solution. Additional punctures were repeated until a visually verified reliable whitish tissue was obtained (ranging from 1 to 5 punctures). At our institution, on-site cytopathological evaluation during the EUS-FNB procedure was not feasible. Instead, we employed a target sample check illuminator to assess the adequacy of tissue acquisition in the biopsy samples [10]. Antithrombotic medications were discontinued in accordance with the guidelines of the Japanese Gastroenterological Endoscopy Society [11]. We did not administer prophylactic antimicrobial agents.

### 2.3. Diagnostic Criteria

**Figure 2:**
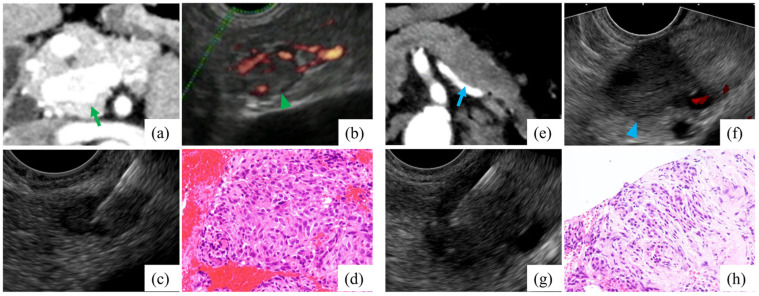
Patient diagnosed as PNEN, hypervascular lesion (**a**–**d**). (**a**) CT scan showing hypervascularity on arterial-phase imaging (green arrow). (**b**) A hypodense lesion with blood vessels on color Doppler endoscopic ultrasonography (green arrowhead). (**c**) EUS-FNB. (**d**) Microscopic appearance of hematoxylin and eosin-stained tissue sample can be observed. Another patient diagnosed with pancreatic ductal adenocarcinoma, hypovascular lesion (**e**–**h**). (**e**) CT scan showing less vascularity on arterial-phase imaging (blue arrow). (**f**) A hypodense lesion without blood vessel on color Doppler endoscopic ultrasonography (blue arrowhead). (**g**) EUS-FNB. (**h**) Microscopic appearance of hematoxylin and eosin-stained tissue sample can be observed. Abbreviations: PNEN, pancreatic neuroendocrine neoplasm; CT, computed tomography; EUS-FNB, endoscopic ultrasound-guided fine-needle aspiration.

The diagnostic accuracy of EUS-FNB was evaluated based on distinguishing malignant lesions from benign diseases. The final diagnosis was based on pathological assessments performed using EUS-FNB, pancreatic juice aspiration cytology, or surgical specimens. Malignant lesions included pancreatic cancers, PNENs, SPNs, metastatic tumors, and malignant lymphomas. Lesions, such as mass-forming pancreatitis, accessory spleens, and other nonneoplastic lesions were regarded as benign. For lesions that were surgically resected, the pathological findings from the surgical specimen were deemed to be the definitive diagnosis. In the case of malignant lesions that did not undergo surgical intervention, the diagnosis was confirmed based on subsequent tumor growth, the emergence of metastases, and the response to chemotherapy. As for benign lesions, a minimum 12-month follow-up was conducted, involving at least two imaging studies, to ascertain their sustained benign nature without any signs of enlargement. While standard clinical follow-up durations typically span at least 6 months, the 12-month timeframe was instituted to more accurately evaluate false-negative outcomes (Figure 2).

### 2.4. Propensity-Score Matching

We conducted propensity-score matching to mitigate the potential impact of between-group variations in the baseline characteristics of our study cohort on the diagnostic accuracy and adverse events associated with EUS-FNB for solid pancreatic lesions. The propensity scores of the patients who underwent EUS-FNB for solid pancreatic hypervascular or hypovascular lesions were computed using a multivariate logistic regression model. Parameters, such as lesion size, location, the use of antithrombotic agents, and needle size have been previously identified as factors influencing diagnosis and adverse events associated with EUS-FNB [4,12,13]. Age, sex, and comorbidities were added, and the following patient characteristics were included in the model: age (continuous), sex (male vs. female), use of antithrombotic agents (yes vs. no), comorbidity (evaluated using the Charlson Comorbidity Index [CCI] [14]), lesion size (continuous), the location of the pancreatic lesion (head vs. body vs. tail), and needle type (19 vs. 22 vs. 25). Employing the nearest-neighbor approach with a caliper range of 0.2 times the standard deviation of pooled propensity scores, each patient in the hypervascular-lesion group was matched to a counterpart in the hypovascular-lesion group.

### 2.5. Adverse Events

Adverse events caused by endoscopic procedures were evaluated and classified according to the American Society for Gastrointestinal Endoscopy guidelines [15].

### 2.6. Statistical Analysis

Statistical analyses were conducted using StatFlex v7.0 for Windows (Artech Co., Ltd., Osaka, Japan). Categorical variables were compared using the chi-square test or Fisher’s exact test, as appropriate, while continuous variables were assessed using the Mann–Whitney U test. All reported values were presented as medians with interquartile ranges. Statistical significance was set at *p* < 0.05.

## 3. Results

### 3.1. Patient Characteristics and Baseline Evaluation

**Figure 3:**
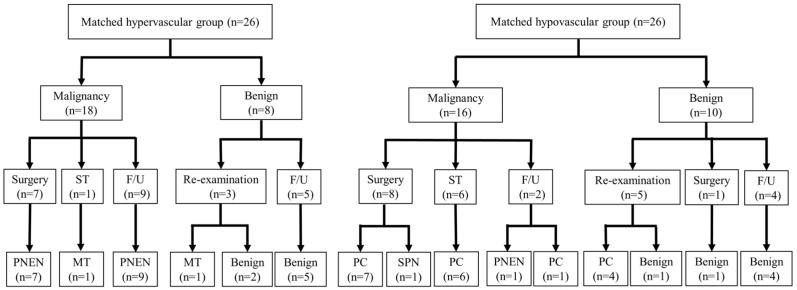
Diagnostic flowchart of the patients in the propensity score-matched cohort. Abbreviations: ST, systemic therapy; F/U, follow-up; PNEN, pancreatic neuroendocrine neoplasm; MT, metastatic tumor; PC, pancreatic cancer; SPN, solid pseudopapillary neoplasm.

A total of 301 participants with 308 solid pancreatic lesions were enrolled in this study. The participants included 172 men and 129 women aged 23–88 years (median, 71 years). Of the lesions, 271 were classified as malignant and 37 were categorized as benign. Specifically, 35 lesions were assigned to the hypervascular group, and 273 lesions were assigned to the hypovascular group. Comprehensive characteristics of all the patients in the hypervascular and hypovascular groups are outlined in Table 1. Several baseline characteristics, including the size and location of the pancreatic lesions and primary disease, were significantly different between the two groups. Through propensity-score matching, these disparities were effectively balanced, resulting in comparable age, sex, pancreatic lesion location, antithrombotic agent use, CCI, and needle size distribution in both groups (Table 2). In the propensity score-matched cohort, the hypervascular group included 19 malignant lesions (17 PNENs and 2 metastatic tumors) and 7 benign lesions (comprising 3 patients with mass-forming pancreatitis and 4 accessory spleens). The hypovascular group included 20 malignant (18 pancreatic cancers, 1 SPN, and 1 PNEN) and 6 benign (comprising 4 patients with mass-forming pancreatitis and 2 other lesions) lesions (Figure 3). The median follow-up period for the benign lesions was 28 months (range, 14–99 months).

### 3.2. Diagnostic Utility of EUS-FNB for Hypervascular Pancreatic Lesions

In the initial cohort prior to propensity score-matching, the diagnostic performance characteristics of EUS-FNB for hypervascular lesions were as follows: sensitivity 95.8% (23/24), specificity 100% (11/11), positive predictive value (PPV) 100% (23/23), negative predictive value (NPV) 91.7% (11/12), and overall accuracy 97.1% (34/35). On the other hand, for hypovascular lesions, the corresponding values were: sensitivity 93.5% (231/247), specificity 100% (26/26), PPV 100% (231/231), NPV 61.9% (26/42), and overall accuracy 94.1% (257/273). Notably, none of these observed differences reached statistical significance.

In the propensity score-matched cohort, the diagnostic yields of EUS-FNB for distinguishing between malignant and benign pancreatic lesions are shown in Table 3. The sensitivity, specificity, PPV, NPV, and overall accuracy of EUS-FNB for hypervascular lesions were 94.7% (18/19), 100% (7/7), 100% (18/18), 87.5% (7/8), and 96.2% (25/26), respectively, and those for hypovascular lesions were 80.0% (16/20), 100% (6/6), 100% (16/16), 60.0% (6/10), and 84.6% (22/26), respectively. Importantly, no substantial disparity was observed in the diagnostic utility of EUS-FNB for hypervascular and hypovascular lesions in the propensity score-matched cohort either (*p* = 0.347).

### 3.3. Adverse Events

Out of the 301 participants, adverse events were experienced by 8 individuals. These adverse events included three cases of bleeding, three cases of pancreatitis, one case of infection, and one case of pain. Within this group, one participant (3.1%) belonged to the hypervascular lesion group, while seven participants (2.6%) were in the hypovascular lesion group. Importantly, there were no statistically significant differences observed between these two groups in terms of the occurrence of adverse events.

In the propensity score-matched cohort, adverse events were reported in a solitary patient (1.9%; pancreatitis in the hypovascular group) (Table 4). There were no adverse events following the EUS-FNB of hypervascular lesions. Notably, no significant differences in adverse events were observed between the hypervascular and hypovascular groups.

## 4. Discussion

Endoscopic ultrasound-guided fine-needle aspiration has gained prominence in the diagnostic assessment of pancreatic lesions, owing to its marked diagnostic efficacy. The sensitivity, specificity, and accuracy of EUS-FNB for solid pancreatic lesions have been reported to be 85.0–90.8%, 96.5–98.0%, and 91.0%, respectively [2,3]. In our investigation, the sensitivity, specificity, and accuracy of EUS-FNB for all solid pancreatic lesions were 93.7%, 100%, and 94.5%, respectively, which aligns with earlier studies.

In the context of this propensity score-matched investigation, EUS-FNB has emerged as a valuable diagnostic tool for pancreatic lesion assessment, irrespective of vascularity. The diagnostic performance for all hypervascular lesions remained robust, with a sensitivity, specificity, and accuracy of 95.8%, 100%, and 97.1%, respectively. A prior study on EUS-FNB for PNEN, which are predominantly hypervascular tumors, reported sensitivity, specificity, and accuracy rates of 94.8%, 99.4%, and 98.7%, respectively [5]. In our study, we obtained similar results. In our propensity score-matched cohort, the sensitivity was slightly reduced in the hypovascular group, but the difference was not significant. Notably, the hypovascular group comprised a substantial proportion of patients with pancreatic cancer. Pancreatic cancer lesions can exhibit indistinct boundaries and are accompanied by pancreatic inflammatory changes, particularly in the tail region. These factors, coupled with lower tumor cell density and heightened fibrosis, could potentially contribute to the observed decline in sensitivity.

Endoscopic ultrasound-guided fine-needle aspiration in cases involving small lesions is widely recognized to pose challenges, encompassing not only the precise target of the lesion but also the acquisition of a sufficient specimen [4,16,17]. Agarwal reported that the sensitivity of EUS-FNB in diagnosing pancreatic lesions with a size of <20 mm was observed to be 75%, in contrast to the sensitivity of 94% recorded for lesions of >20 mm [16]. Hwang reported that accuracies were determined to be 71% for lesions <20 mm and 90% for lesions >30 mm [17]. Conversely, some studies reported high sensitivity, regardless of lesion size [18,19]. In our investigation, the size in three of five false-negative patients was <30 mm, and the sensitivity was slightly lower for ≤20 mm lesions than that for >20 mm lesions (91.7% vs. 94.8%, *p* = 0.300; not presented in the Results). Although no statistically significant difference was observed, it is plausible that lesion size could potentially impact diagnostic performance. Notably, lesions located in the pancreatic body or tail have been documented to exhibit more accurate diagnoses than those located in the pancreatic head [4]. Thus, the EUS-FNB of lesions positioned within the pancreatic head may present challenges. This is because of the pronounced angulation and torque applied to the needle when approaching lesions in this region [19]. In our study, the location of lesions did not affect the diagnostic accuracy (accuracies for head, body, and tail lesions were 94.9%, 90.2%, and 100%, respectively; not presented in the Results).

Considering the information presented above, we hypothesize that lesion size may indeed influence the diagnostic performance of EUS-FNB. Furthermore, while not explicitly explored in this study, the proficiency of the endoscopist might also play a role in diagnostic outcomes. It is worth noting that we were unable to assess the potential impact of on-site cytopathological evaluation. Conversely, the target sample check illuminator, which was utilized for all punctures, might contribute to various factors influencing the examination results, although its specific utility could not be evaluated [2,4,10].

The incidence of EUS-FNB-related adverse events has been reported to be low (1.7–4.1%) [8,20,21,22]. In our investigation, the occurrence rate of post-procedural adverse events across all lesions (3.2%) exhibited similarity or a slight elevation in comparison to findings from prior investigations. Of 301 participants, adverse events were encountered in 8 individuals. Notably, only one patient had a severe infection requiring surgical intervention, whereas the remaining patients were characterized by mild-to-moderate events.

Common adverse events associated with EUS-FNB include bleeding and pancreatitis [22]. The incidence of adverse events associated with EUS-FNB for pancreatic hypervascular lesions was considered to be relatively elevated in comparison to that for pancreatic hypovascular lesions, particularly in relation to bleeding. Nevertheless, our study did not show discernible differences in the occurrence of adverse events between patients with hypervascular and hypovascular pancreatic lesions. In a study by Katanuma et al., small lesions (≤20 mm) and PNEN were risk factors for adverse events following EUS-FNB [8]. Smaller lesions may pose greater challenges to the passage of needles than larger lesions. This may result in damage to the pancreatic parenchyma and cause adverse events, such as pancreatitis and bleeding. In general, PNENs are hypervascular tumors, and the risk of bleeding from these tumors may be higher than that from other tumors. Furthermore, PNENs exhibit other distinctive features, such as diminutive size and normal adjacent pancreatic parenchyma. These factors may necessitate more needle traversals through the normal pancreatic tissue, which may amplify the risk of adverse events. In our study, propensity-score matching may have been able to evaluate the risk by focusing on lesion vascularity.

In general, antithrombotic medications are considered risk factors for EUS-FNB, especially bleeding [11]. Recently, several guidelines have been published on the management of patients receiving antithrombotic treatments during endoscopic procedures [10,23,24]. It has been established that EUS-FNB can be conducted safely when adhering to these stipulations [25]. Moreover, the presence of comorbidities is a potential risk factor of adverse events associated with endoscopic procedures [26]. For EUS-FNB, the rate of adverse events has been reported to be high in patients with cirrhosis [27]. However, few studies have investigated the potential correlation between comorbidities and the likelihood of adverse events. Although the possibility of cardiac or renal conditions serving as risk factors for adverse events during EUS-FNB has been considered, this remains uncertain. From a theoretical perspective, the use of larger-diameter needles for EUS-FNB is expected to correlate with an increased incidence of adverse events. Needle passes using large-diameter needles can increase tissue damage during insertion. Although some reports have suggested that 25 G needles may cause fewer adverse events [22,25], a meta-analysis reported no difference between 22 G and 25 G needles in terms of adverse event rate [28]. Some reports have suggested that a higher number of punctures and increased to and fro movements may increase the risk of adverse events due to increased tissue damage [29,30]. However, evaluating this aspect may prove challenging because of potential skill-related biases among endoscopists. In this investigation, by meticulously matching factors potentially linked to adverse events, we posit that EUS-FNB is a reliable procedure, irrespective of vascularity.

This study has certain limitations. First, this was a single-center retrospective study with a relatively small cohort, particularly for patients with hypovascular lesions. Being devoid of prospective randomization, this study could not entirely eliminate the impact of unaccounted factors that may be related to diagnostic efficacy and occurrence of adverse events during EUS-FNB. Secondly, our categorization of pancreatic lesions as hypervascular was based on contrast enhancement during the arterial and portal phases of dynamic CT. It is possible that some tumors were actually hypervascular, but were labeled as hypovascular because of the timing of imaging. Additionally, individuals who were unable to undergo dynamic CT, such as those with severe chronic kidney disease with a potential risk of bleeding, may have been excluded from the study. The effectiveness of contrast-enhanced EUS has been a subject of recent investigation [31]. While we did not employ contrast prior to EUS-FNB in our study, it is conceivable that the inclusion of contrast may enable a more accurate assessment of vascularity and guide the procedure. Third, the frequency of adverse events may depend on the expertise of the endoscopist; however, this aspect was not assessed. Technical disparities in EUS-FNB may arise due to the proficiency of each endoscopist. Recently, newer end cutting EUS-FNB needles have been launched and the utility has been documented [32,33]; however, given the limited usage of these needles in our study, distinguishing between different needle types was unfeasible. Furthermore, we assessed diagnostic accuracy predominantly through clinical follow-up, which enabled us to evaluate accuracy in a binary classification framework, primarily distinguishing between malignant and benign cases. However, the assessment of multiclass accuracy was not feasible in our study due to the nature of our diagnostic definitions. To comprehensively determine whether the vascularity of pancreatic lesions correlates with diagnostic efficacy and incidence of adverse events, a large-scale multicenter prospective study focused on surgically resected pancreatic lesions and employing uniform techniques and needle types is warranted.

## 5. Conclusions

No discernible differences were observed in the diagnostic efficacy or safety of EUS-FNB when applied to solid pancreatic lesions, irrespective of their hypervascular or hypovascular nature. This implies that EUS-FNB may exhibit not only robust diagnostic accuracy for solid pancreatic lesions but also a commendable level of safety, regardless of the vascularity of the lesions.

## Figures and Tables

**Table 1:** Baseline characteristics of the study patients.

	Hypervascular Group (*n* = 35)	Hypovascular Group (*n* = 273)	*p*-Value
Age, median (range, years)	71 (34–84)	71 (23–88)	0.416
Sex, male/female	21/11	151/118	0.305
Size, median (range, mm)	9 (5–45)	27 (5–110)	<0.001
Location of pancreatic lesion			
Head/Body/Tail	7/14/14	129/89/55	0.004
Antithrombotic agents (yes/no)	4/28	29/241	0.993
Comorbidities			0.798
Myocardial infarction	0	9	
Congestive heart failure	0	5	
Peripheral vascular disease	2	3	
Cerebrovascular disease	3	16	
Dementia	1	1	
Chronic pulmonary disease	0	10	
Rheumatoid disease	0	1	
Peptic ulcer	2	32	
Mild liver disease	3	15	
Diabetes without chronic adverse event	7	58	
Hemiplegia or paraplegia	0	5	
Moderate or severe renal disease	0	1	
Diabetes with chronic adverse event	0	2	
Tumor without metastases	7	53	
Leukemia	0	0	
Lymphoma	0	4	
Moderate or severe liver disease	0	1	
Metastatic solid tumor	0	3	
AIDS	0	0	
Needle (G)			
19/22/25/Multiple/Unknown	0/23/11/1/0	20/170/73/4/6	0.404
Primary disease			
Malignant	(24)	(247)	<0.001
Pancreatic cancer	0	231	
ITPN	0	1	
PNEN	22	6	
SPN	0	7	
Metastatic tumor	2	1	
Malignant lymphoma	0	1	
Benign	(11)	(26)	
Mass-forming pancreatitis	3	24	
Accessory spleen	8	0	
Sarcoidosis	0	1	
Fatty metamorphosis	0	1	
Tumor marker			
CEA, median (range, ng/mL)	1.8 (0.8–7.2)	3.5 (0.8–986.6)	<0.001
CA19-9, median (range, U/mL)	11.5 (0.8–221.8)	107.1 (0.8–20,410)	<0.001
SPan-1, median (range, U/mL)	8.2 (1.0–71.0)	63.0 (1.0–60,000)	<0.001
DUPAN-2, median (range, U/mL)	25.0 (25.0–264.0)	146.0 (25.0–101,000)	<0.001
Number of punctures, median (range)	2 (1–4)	2 (1–5)	0.486

Abbreviations: AIDS, acquired immunodeficiency syndrome; ITPN, intraductal tubulopapillary neoplasm; PNEN, pancreatic neuroendocrine neoplasm; SPN, solid pseudopapillary neoplasm; CEA, carcinoembryonic antigen; CA19-9, carbohydrate antigen 19-9; SPan-1, s-pancreas antigen-1; DUPAN-2, pancreatic cancer-associated antigen-2.

**Table 2:** Baseline characteristics of the propensity score-matched cohort.

	Hypervascular Group (*n* = 26)	Hypovascular Group (*n* = 26)	*p*-Value
Age, median (range, years)	74 (34–84)	70 (49–83)	0.714
Sex, male/female	16/10	10/16	1.000
Size, median (range, mm)	12.3 (5.0–45.0)	13.0 (5.0–45.0)	0.557
Location of pancreatic lesion			
Head/Body/Tail	7/13/6	6/13/7	0.926
Antithrombotic agents (yes/no)	2/24	2/24	1.000
CCI, median (range)	1 (0–3)	1 (0–4)	0.615
Disease			
Malignant			
Pancreatic cancer	0	18	
PNEN	17	1	
SPN	0	1	
Metastatic tumor	2	0	
Benign			
Mass-forming pancreatitis	3	4	
Accessory spleen	4	0	
Others	0	2	
Tumor marker			
CEA, median (range, ng/mL)	1.75 (0.8–7.2)	2.3 (1.0–30.0)	0.136
CA19-9, median (range, U/mL)	10.55 (0.8–211.8)	23.8 (0.8–1591.6)	0.669
SPan-1, median (range, U/mL)	6.8 (1.0–71.0)	18.5 (1.0–220.0)	0.007
DUPAN-2, median (range, U/mL)	25.0 (25.0–264.0)	90.5 (25.0–11,800)	<0.001
Needle (G)			
22/25/Multiple	17/8/1	12/14/0	0.672
Number of punctures, median (range)	2 (1–4)	2 (1–5)	0.881

Abbreviations: CCI, Charlson Comorbidity Index; PNEN, pancreatic neuroendocrine neoplasm; SPN, solid pseudopapillary neoplasm; CEA, carcinoembryonic antigen; CA19-9, carbohydrate antigen 19-9; SPan-1, S-pancreas antigen-1; DUPAN-2, pancreatic cancer-associated antigen-2.

**Table 3:** Diagnostic performance in the propensity score-matched cohort.

	Hypervascular Group (*n* = 26)	Hypovascular Group (*n* = 26)	*p*-Value
Diagnosis			
Sensitivity, %	94.7 (18/19)	80.0 (16/20)	0.370
Specificity, %	100 (7/7)	100 (6/6)	1.000
PPV, %	100 (18/18)	100 (16/16)	1.000
NPV, %	87.5 (7/8)	60.0 (6/10)	0.444
Accuracy, %	96.2 (25/26)	84.6 (22/26)	0.347

Abbreviations: PPV, positive predictive value; NPV, negative predictive value.

**Table 4:** Adverse events in the propensity score-matched cohort.

	Hypervascular Group (*n* = 26)	Hypovascular Group (*n* = 26)	*p*-Value
Cardiovascular	0	0	1.000
Pulmonary	0	0	1.000
Thromboembolic	0	0	1.000
Instrumental	0	0	1.000
Bleeding	0	0	1.000
Infection	0	0	1.000
Drug reaction	0	0	1.000
Pain	0	0	1.000
Pancreatitis	0	1	1.000
Integument	0	0	1.000
Other	0	0	1.000
All	0	1	1.000

## Data Availability

The datasets generated and/or analyzed in the current study are available from the corresponding author upon reasonable request.
